# Correlation Between Systemic Inflammation, Gut Microbiome Dysbiosis and Postoperative Complications After the Modified Whipple Procedure

**DOI:** 10.3390/biomedicines13010104

**Published:** 2025-01-05

**Authors:** Gelu Mihai Breaza, Florin Emil Hut, Octavian Cretu, Simona-Alina Abu-Awwad, Ahmed Abu-Awwad, Laurențiu Vasile Sima, Radu Gheorghe Dan, Cristina Ana-Maria Dan, Raluca Maria Closca, Flavia Zara

**Affiliations:** 1 Doctoral School, “Victor Babes” University of Medicine and Pharmacy, 300041 Timisoara, Romania; gelu.breaza@umft.ro; 2 University Clinic of Surgery I, “Victor Babes” University of Medicine and Pharmacy, 300041 Timisoara, Romania; octavian.cretu@umft.ro (O.C.); sima.laurentiu@umft.ro (L.V.S.); radu.dan@umft.ro (R.G.D.); cristina.dan@umft.ro (C.A.-M.D.); 3 Center for Hepato-Bilio-Pancreatic Surgery, “Victor Babes” University of Medicine and Pharmacy, 300041 Timisoara, Romania; 4 Ist Clinic of Obstetrics and Gynecology, “Pius Brinzeu” County Clinical Emergency Hospital, 300723 Timisoara, Romania; alina.abuawwad@umft.ro; 5 Department of Obstetrics and Gynecology, Faculty of Medicine, “Victor Babes” University of Medicine and Pharmacy, 300041 Timisoara, Romania; 6 Department XV—Discipline of Orthopedics—Traumatology, “Victor Babes” University of Medicine and Pharmacy, 300041 Timisoara, Romania; ahm.abuawwad@umft.ro; 7 Research Center University Professor Doctor Teodor Sora, “Victor Babes” University of Medicine and Pharmacy, 300041 Timisoara, Romania; 8 Department of Pathology, Emergency City Hospital, 300254 Timisoara, Romania; raluca.moaca@umft.ro (R.M.C.); flavia.zara@umft.ro (F.Z.); 9 Department of Microscopic Morphology, University of Medicine and Pharmacy “Victor Babes”, 300041 Timisoara, Romania

**Keywords:** pancreatic head cancer, modified Whipple procedure, perioperative complications, immunological markers, pancreatic fistula, delayed gastric emptying, CRP, IL-6, intestinal microbiome

## Abstract

(1) Background: The modified Whipple procedure, or pylorus-preserving pancreaticoduodenectomy, is a complex surgical intervention used to treat pancreatic head tumors. While preserving digestive function, it is associated with significant perioperative risks. This study explores the clinical, immunological, and microbiome-related factors influencing postoperative complications, focusing on the interplay between patient comorbidities, systemic inflammation, and gut dysbiosis. (2) Methods: A retrospective analysis was conducted on 123 patients undergoing the modified Whipple procedure for pancreatic head tumors. Patients were categorized into two groups based on the occurrence of significant postoperative complications (Group A: with complications; Group B: without complications). Data on demographics, comorbidities, inflammatory markers (CRP, IL-6, procalcitonin), and gut microbiome composition were collected. Microbial diversity was evaluated using the Shannon Index, and logistic regression was performed to identify independent predictors of complications. (3) Results: Patients in Group A had a significantly higher prevalence of diabetes mellitus (43.1% vs. 20.8%; *p* = 0.02) and cardiovascular disease (35.3% vs. 13.9%; *p* = 0.01). Elevated inflammatory markers (CRP ≥ 40 mg/L, IL-6 ≥ 30 pg/mL, procalcitonin ≥ 0.5 ng/mL) were strongly associated with higher complication rates. Microbiome analysis indicated dysbiosis in Group A, with reduced *Lactobacillus* and *Bifidobacterium* levels, increased *Enterobacteriaceae* abundance, and a lower Shannon Index (<2). Patients exhibiting both dysbiosis and elevated inflammation had the highest complication rate (60%). Multivariate analysis identified diabetes, elevated IL-6, and dysbiosis as independent predictors of adverse outcomes. (4) Conclusions: Postoperative complications after the modified Whipple procedure are influenced by systemic inflammation and gut dysbiosis. A systematic preoperative assessment of microbiome health and inflammatory markers enables accurate risk stratification and personalized interventions, potentially reducing the incidence of complications and improving overall surgical outcomes.

## 1. Introduction

Pancreatic cancer, particularly ductal adenocarcinoma localized in the head of the pancreas, remains one of the most challenging malignancies to treat due to its aggressive progression, late diagnosis, and poor prognosis [1]. Despite advances in diagnostic and therapeutic techniques, the majority of patients present with advanced disease, limiting the efficacy of curative interventions [2]. Surgical resection, specifically pancreaticoduodenectomy (Whipple procedure), remains the cornerstone of curative therapy for pancreatic head cancer [3]. However, this complex surgery carries a high risk of perioperative complications, including pancreatic fistula, hemorrhage, delayed gastric emptying, and infections, which contribute to significant morbidity, prolonged hospital stays, and delayed recovery [4].

The modified Whipple procedure, or pylorus-preserving pancreaticoduodenectomy, was developed to address the functional disadvantages associated with the traditional Whipple technique. By preserving the pylorus and a portion of the stomach, the modified procedure aims to reduce gastrointestinal complications, such as delayed gastric emptying, and improve long-term nutritional outcomes and quality of life [5]. While this approach offers functional benefits, perioperative complications remain a major concern, and the underlying factors influencing these outcomes are not yet fully understood [6].

Emerging evidence suggests that perioperative complications are strongly influenced by the interplay between systemic inflammation, gut microbiome health, and patient-specific factors. Elevated inflammatory markers, such as C-reactive protein (CRP), interleukin-6 (IL-6), and procalcitonin, have been consistently associated with poor postoperative outcomes, reflecting an exaggerated immunological response to surgical trauma [7]. At the same time, disruptions in the gut microbiome—characterized by reduced microbial diversity and a shift towards pathogenic taxa—have been implicated in exacerbating systemic inflammation, potentially creating a synergistic effect that worsens postoperative recovery [8]. These findings highlight the need for a deeper understanding of how inflammation and microbiome composition interact to influence perioperative outcomes.

In this context, the modified Whipple procedure, though oncological effective and less disruptive to gastrointestinal continuity, provides a valuable framework for exploring these factors. By examining differences in microbiome composition, inflammatory markers, and their association with complications, this study aims to uncover key insights into the mechanisms driving adverse outcomes.

This study’s objective is to evaluate the relationship between gut microbiome composition, systemic inflammatory markers, and perioperative complications in patients undergoing the modified Whipple procedure for pancreatic head cancer. By comparing patients with and without complications, this study seeks to elucidate the role of dysbiosis, systemic inflammation, and their interaction in influencing surgical outcomes. The findings aim to inform targeted strategies for perioperative management, ultimately reducing complication rates and improving the overall prognosis and recovery of patients undergoing pancreatic surgery.

## 2. Materials and Methods

This retrospective study evaluated the interplay between clinical factors, gut microbiome composition, and systemic inflammatory markers about perioperative complications among patients undergoing the modified Whipple procedure (pylorus-preserving pancreaticoduodenectomy) for pancreatic ductal adenocarcinoma localized in the head of the pancreas. The analysis encompassed ten years, including patients treated at Timişoara Municipal Emergency Clinical Hospital between 2013 and 2023.

### 2.1. Study Design and Population

A total of 123 patients who underwent the modified Whipple procedure were included in the study. Patients were categorized into two groups based on perioperative outcomes. Group A consisted of 51 patients (41%) who developed documented perioperative complications within 30 days post-surgery, including pancreatic fistula, delayed gastric emptying, postoperative bleeding, infections, or other significant adverse events. Group B consisted of 72 patients (59%) who did not experience any documented perioperative complications. The surgeries were performed by multiple surgical teams, with composition changing over the study period. Despite these changes, all surgeons involved had extensive experience in pancreatic surgery, with a minimum of 10 years of practice and full proficiency in performing the modified Whipple procedure. The surgical technique remained consistent across all cases, strictly adhering to the principles of the pylorus-preserving pancreaticoduodenectomy, including standardized steps for tumor resection and digestive tract reconstruction. Perioperative and postoperative management protocols, including antibiotic prophylaxis, nutritional support, pain management, and complication monitoring, were standardized and applied uniformly throughout the study period, with minor updates reflecting advancements in clinical care. These measures ensured high consistency and comparability in surgical and clinical outcomes across the study population. The study population was further analyzed to explore the influence of gut microbiome composition and systemic inflammatory markers on the observed outcomes, focusing on the potential interaction between dysbiosis and inflammation.

### 2.2. Data Collection

Clinical and demographic data were extracted from patient medical records, including age, sex, and body mass index (BMI). Comorbidities such as diabetes mellitus, cardiovascular disease, and other relevant conditions were also recorded. Tumor characteristics, including size, stage, and histological type, were documented to provide a detailed overview of the study population. Gut microbiome composition was assessed using preoperative stool samples analyzed for microbial diversity, measured using the Shannon Index, and bacterial taxa abundance determined through 16S rRNA sequencing. Particular attention was given to the relative abundance of *Lactobacillus* and *Bifidobacterium* as beneficial bacteria, and *Enterobacteriaceae* and *Clostridioides* difficile as pathogenic taxa.

Inflammatory markers, including C-reactive protein (CRP), interleukin-6 (IL-6), procalcitonin, fibrinogen, and D-dimer, were measured at four distinct time points: preoperatively and at 24, 48, and 72 h postoperatively. The primary outcome of the study was the incidence and type of perioperative complications. In contrast, secondary outcomes included the duration of hospital stay, 30-day mortality, and the relationships between gut microbiome status, systemic inflammatory markers, and perioperative outcomes. This comprehensive dataset facilitated an in-depth analysis of the interplay between microbiome imbalances, systemic inflammation, and adverse postoperative events, offering valuable insights into the mechanisms driving surgical complications.

### 2.3. Surgical Technique

All patients in this study underwent the modified Whipple procedure, a surgical technique also referred to as pylorus-preserving pancreaticoduodenectomy. This highly intricate and extensive operation aims to remove tumors localized in the head of the pancreas while preserving critical structures to maintain as much of the digestive system’s normal function as possible. The procedure involves the precise removal of the pancreatic head, which is the site of the tumor. Along with this portion of the pancreas, the surgery also entails excising several adjacent structures to ensure the complete removal of cancerous tissues. These include the entire duodenum, the initial segment of the small intestine, a portion of the proximal jejunum, the next section of the small intestine, the gallbladder, and a segment of the bile duct. This comprehensive approach is essential to minimize the risk of residual malignant tissue while optimizing postoperative outcomes.

A vital aspect of this modified procedure is the preservation of the pylorus, the valve that controls the passage of food from the stomach into the small intestine. Unlike the traditional Whipple procedure, which involves the removal of the distal stomach, the modified technique retains the stomach and its pyloric function, aiming to reduce complications such as delayed gastric emptying and to maintain better postoperative nutritional status.

After removing the necessary organs and tissues, the digestive tract must be reconstructed to ensure normal digestive function. This is achieved through a series of precise surgical connections: an end-to-side pancreaticojejunostomy, in which the remaining pancreas is connected to the jejunum to allow pancreatic enzymes to enter the small intestine; a choledochojejunostomy, where the bile duct is connected to the jejunum to restore bile flow; and a gastrojejunostomy, in which the stomach is connected directly to the jejunum, bypassing the resected duodenum. This complex reconstruction allows the digestive system to continue functioning, even after removing several vital structures [9,10].

### 2.4. Inclusion and Exclusion Criteria

Inclusion criteria:

- Patients diagnosed with pancreatic cancer localized in the head of the pancreas, confirmed by imaging and histopathology, and deemed resectable.
- Patients who underwent the modified Whipple procedure (pylorus-preserving pancreaticoduodenectomy).
- Age between 18 and 85 years.
- ASA physical status classification I or II.
- Availability of preoperative stool samples for gut microbiome analysis.
- Complete medical records and postoperative monitoring for up to 30 days.
- Operable tumors confined solely to the head of the pancreas, with no major vascular invasion or distant metastases.
- Patients who provided informed consent for inclusion in the retrospective study.

Exclusion criteria:

- Patients with locally advanced or metastatic pancreatic tumors deemed unresectable by the surgical team.
- Previous major abdominal surgeries that could alter the anatomical structures involved in the Whipple procedure or affect microbiome analysis.
- Recent use of antibiotics, probiotics, or other treatments that could impact gut microbiome composition.
- Presence of concurrent malignancies requiring additional surgical or oncological treatment, which could confound perioperative outcomes.
- Preoperative systemic infections or sepsis, which could independently influence postoperative complications.
- Incomplete medical records or insufficient postoperative monitoring to evaluate complications accurately.
- Pregnancy or lactation, due to distinct physiological considerations and risks associated with surgical procedures.

### 2.5. Statistical Analysis

All statistical analyses were conducted using GraphPad Prism 6 software to evaluate the associations between demographic, clinical, microbiome, and inflammatory variables and perioperative outcomes. Continuous variables, such as age, BMI, inflammatory marker levels (CRP, IL-6, procalcitonin, fibrinogen, and D-dimer), and microbial diversity index (Shannon), were expressed as mean ± standard deviation (SD). Normality was assessed using the Shapiro–Wilk test. Depending on the distribution, comparisons between groups were made using Student’s *t*-test for normally distributed data or the Mann–Whitney U test for non-normally distributed data.

Categorical variables were expressed as frequencies and percentages, including complication rates and microbiome composition (presence of specific bacterial taxa). Depending on expected cell counts, group comparisons for categorical variables were analyzed using the chi-square test or Fisher’s exact test.

A two-step logistic regression analysis was performed to identify independent predictors of perioperative complications. A multivariate logistic regression model included variables with a *p*-value < 0.05 in univariate analyses. This model evaluated the predictive impact of clinical comorbidities (e.g., diabetes mellitus, cardiovascular disease), inflammatory markers, and microbiome-related factors (e.g., *Lactobacillus* abundance, Shannon Index) on postoperative complications. The results of logistic regression analyses were presented as odds ratios (OR) with corresponding 95% confidence intervals (CI).

All *p*-values were two-sided, and the statistical significance was set at *p* < 0.05. Data were analyzed to examine the interrelationships between gut microbiome dysbiosis, systemic inflammation, and perioperative complications. We also explored the synergistic effects between microbiome imbalance and elevated inflammatory markers on adverse surgical outcomes.

### 2.6. Ethical Consideration

This study was conducted under the ethical standards outlined in the Declaration of Helsinki. Before the data collection, ethical approval was obtained from the institutional review board (IRB) of Timişoara Municipal Emergency Clinical Hospital. Given the study’s retrospective nature, the IRB waived informed consent; however, patient confidentiality was strictly maintained. All data were anonymized to ensure privacy and prevent the identification of individual patients. The study posed no direct risks to participants, as it involved the analysis of existing medical records without any intervention or direct patient contact.

## 3. Results

Table 1 outlines the baseline demographic and clinical characteristics of the study groups. While there were no significant differences in age, sex, or BMI, comorbidities such as diabetes mellitus and cardiovascular disease were significantly more prevalent in Group A. These comorbidities may act as confounding factors, potentially influencing systemic inflammation and microbiome composition.

Table 2 below presents a comprehensive analysis of the distribution of major postoperative complications observed in Group A, providing a detailed insight into their frequency and clinical relevance.

Table 3 highlights significant differences in the gut microbiome composition between Groups A and B. Group A showed marked microbiome dysbiosis, characterized by a substantial reduction in beneficial bacteria (*Lactobacillus*, *Bifidobacterium*) and an increase in pathogenic taxa (*Enterobacteriaceae*, *Clostridioides* difficile). The Shannon Index further confirmed the reduced microbial diversity in this group, suggesting disrupted gut balance.

Table 4 demonstrates a clear association between gut microbiome status and systemic inflammatory markers. Patients with dysbiosis exhibited significantly higher levels of CRP, IL-6, and procalcitonin, indicating systemic immune activation. Elevated fibrinogen and D-dimer levels suggest a heightened risk of thrombotic complications in this group.

Table 5 illustrates the relationship between elevated inflammatory marker levels and the incidence of perioperative complications. Higher levels of inflammatory markers were consistently associated with increased complication rates. IL-6 and procalcitonin demonstrated the strongest predictive value, with complication rates exceeding 55% in patients with elevated levels. These findings emphasize the predictive value of inflammatory markers in identifying patients at greater risk for adverse postoperative outcomes. All differences were statistically significant, reinforcing the association between systemic inflammation and complication severity.

Table 6 highlights the combined influence of gut microbiome status and systemic inflammation on perioperative complication rates. Patients with dysbiosis and high inflammation had the highest complication rates, highlighting a synergistic relationship. Reduced inflammation without dysbiosis was associated with the lowest complication rates. These findings suggest that both microbiome status and inflammation independently and interactively contribute to adverse surgical outcomes, with statistically significant differences across all groups.

**Figure 1:**
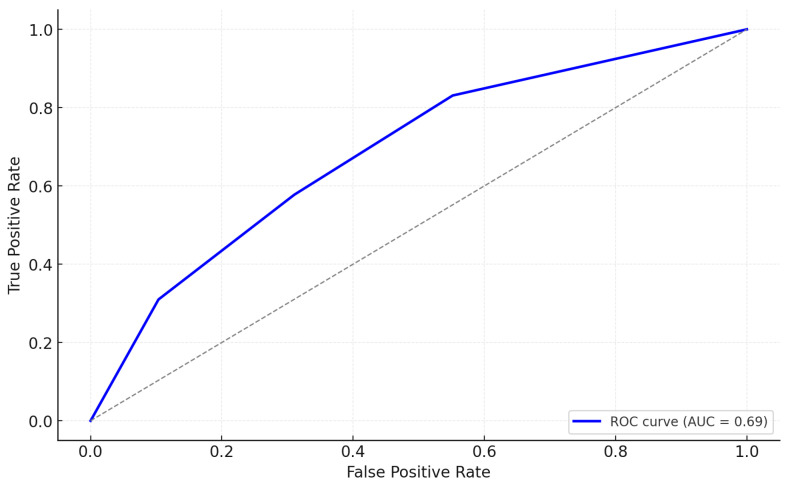
ROC curve for logistic regression analysis of dysbiosis and inflammation on perioperative complications.

Table 7 presents the results of a logistic regression analysis evaluating the combined effects of dysbiosis and inflammation on the likelihood of perioperative complications. Patients with both dysbiosis and high inflammation had the highest risk, with an odds ratio (OR) of 7.4 (95% CI: 4.0–13.8, *p* < 0.001), indicating a 7.4-fold increased likelihood of complications compared to the reference group (no dysbiosis and low inflammation). Dysbiosis combined with low inflammation also significantly increased the risk (OR: 2.9, 95% CI: 1.5–5.8, *p* = 0.01), as did high inflammation in the absence of dysbiosis (OR: 3.2, 95% CI: 1.8–5.7, *p* = 0.003). These findings underscore the synergistic relationship between gut microbiome imbalances and systemic inflammation in driving adverse perioperative outcomes. Figure 1 represents the ROC curve for these findings.

**Figure 2:**
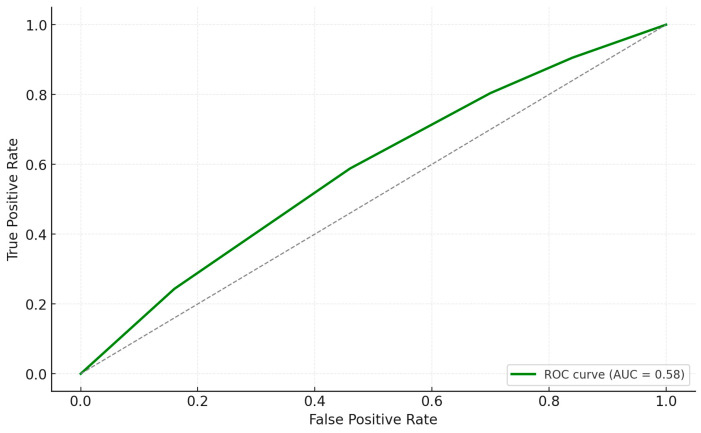
ROC curve for logistic regression analysis of risk factors associated with perioperative complications.

Elevated IL-6 (≥30 pg/mL), CRP (≥40 mg/L), procalcitonin (≥0.5 ng/mL), and microbiome-related factors such as a reduced Shannon Index (<2), low *Lactobacillus* levels (<25%), and increased *Enterobacteriaceae* abundance (>30%) were identified as the most significant predictors of perioperative complications (Table 8). Among comorbidities, diabetes mellitus emerged as an independent risk factor, while cardiovascular disease was not statistically significant (*p* = 0.07). Inflammatory markers consistently increased the odds of complications, with IL-6 demonstrating the strongest association (OR 4.5). Furthermore, microbiome dysbiosis, particularly a diminished Shannon Index and reduced *Lactobacillus* levels, was strongly associated with adverse outcomes, underscoring the critical role of microbiome health in influencing surgical recovery. Figure 2 represents the ROC curve for these findings.

## 4. Discussion

This study provides a comprehensive analysis of the clinical, immunological, and microbiome-related factors associated with perioperative complications in patients undergoing the modified Whipple procedure for pancreatic head cancer. The findings highlight the interplay between patient characteristics, surgical outcomes, and underlying biological mechanisms.

The baseline characteristics demonstrated no significant differences in age, sex, or BMI between the two groups, ensuring demographic comparability. However, Group A had a significantly higher prevalence of comorbidities, particularly diabetes mellitus and cardiovascular disease. Multivariate analysis identified diabetes mellitus as an independent predictor of complications, consistent with evidence linking it to impaired immune function and delayed wound healing [11]. In contrast, cardiovascular disease, despite its higher prevalence in Group A, did not reach statistical significance, aligning with studies suggesting its effects are indirect or overshadowed by dominant factors such as systemic inflammation [12].

This higher burden of comorbidities in Group A directly impacted postoperative outcomes, including the length of hospital stay.

The length of hospital stay was significantly longer in Group A (18.4 ± 4.6 days) compared to Group B (10.2 ± 2.7 days). This result underscores the profound impact of postoperative complications on recovery time and healthcare resource utilization. Similar findings have been reported in other studies, where patients experiencing complications after pancreaticoduodenectomy required extended hospitalization, with stays ranging from 16 to 22 days for complicated cases [13]. These observations emphasize the need for refining patient selection criteria, optimizing surgical techniques, and enhancing postoperative care protocols to mitigate complications in this high-risk population.

The overall complication rate in this study aligns with the literature, where complications following the modified Whipple procedure typically range from 30% to 50% [14]. However, the incidence of pancreatic fistula in our cohort (47%) was notably higher than the commonly reported rates of 20–40% [15], suggesting that our patient population may have had a higher risk profile or subtle differences in surgical technique. Similarly, the delayed gastric emptying rate (21.6%) was consistent with reported values of 20–40% [16], reflecting the expected challenges in gastrointestinal recovery post-surgery.

Postoperative infections occurred in 17.6% of Group A patients. While this rate is within the reported range [17], it highlights the importance of meticulous intraoperative and postoperative management. Early identification of at-risk patients through markers such as diabetes mellitus [18] and elevated inflammatory markers could guide targeted preoperative and postoperative strategies to reduce these risks.

Intraoperative bleeding was observed in 13.7% of Group A cases, within the range reported in the literature (5–16%) [19]. This finding reflects the inherent technical complexity of the modified Whipple procedure and underscores the importance of vigilant intraoperative management to minimize this severe complication.

Biliary fistulas, although less common (5.9%), contribute significantly to overall morbidity. Their incidence aligns with reported rates of 3–8% following pancreaticoduodenectomy [20]. While often benign, biliary fistulas can occasionally progress to life-threatening complications, emphasizing the need for precision in biliary reconstruction and vigilant postoperative care. Intestinal complications, such as fistulas (3.9%) and obstructions (7.8%), also presented notable challenges, frequently necessitating additional interventions and extended hospitalization.

Beyond clinical and demographic factors, microbiome composition emerged as a crucial determinant of postoperative complications. Patients in Group A exhibited marked dysbiosis, characterized by reduced *Lactobacillus* and *Bifidobacterium* levels, increased pathogenic taxa (e.g., *Enterobacteriaceae* and *Clostridioides* difficile), and a lower Shannon Index. Dysbiosis is increasingly recognized as a driver of surgical morbidity. For instance, one study reported that disrupted gut microbiota amplifies systemic inflammation and compromises barrier integrity [21]. In our cohort, reduced microbial diversity (Shannon Index < 2) independently predicted complications, with an odds ratio (OR) of 5.4. These findings suggest the therapeutic potential of microbiome-targeted interventions, such as probiotics, in reducing adverse outcomes.

Emerging evidence underscores the pivotal role of the gut microbiota in modulating systemic inflammation and influencing surgical outcomes [22]. Interventions targeting the microbiome, such as fecal microbiota transplantation (FMT), have shown promise in restoring microbial balance and reducing inflammation. Studies have demonstrated that FMT can significantly improve microbial diversity and the abundance of beneficial taxa such as *Lactobacillus* and *Bifidobacterium* while decreasing pathogenic bacteria like *Enterobacteriaceae* and *Clostridioides* difficile [23]. These microbiome-modulating strategies could mitigate postoperative complications in high-risk surgical populations, including those undergoing pancreaticoduodenectomy. Future research should explore the integration of FMT into perioperative care protocols to enhance recovery and reduce adverse outcomes.

Systemic inflammation also played a central role in postoperative complications. Elevated preoperative levels of CRP (>10 mg/L) and IL-6 (>30 pg/mL) were significant predictors of adverse outcomes, with odds ratios of 3.4 and 4.2, respectively. These findings align with prior research linking inflammation to impaired healing and increased susceptibility to infections [24,25,26].

In addition to its effects on the microbiome, FMT may also regulate systemic inflammation by reducing gut barrier permeability and endotoxemia [27], key drivers of inflammatory responses post-surgery. By addressing the interplay between dysbiosis and inflammation, FMT could serve as a dual-action therapeutic approach, targeting two major contributors to postoperative morbidity. Such interventions align with the broader movement toward personalized medicine, where microbiome-targeted therapies are tailored to individual risk profiles to optimize outcomes.

Procalcitonin, a well-established marker of bacterial infections [28], was particularly useful in identifying patients at risk for sepsis and other infectious complications. Elevated procalcitonin levels (>0.5 ng/mL) have consistently been associated with heightened risks of sepsis and infections in surgical patients [29]. Similarly, abnormal coagulation parameters, such as elevated fibrinogen and D-dimer levels, were linked to an increased risk of thromboembolic complications, underscoring the importance of early identification and targeted interventions [30].

The findings of this study underscore the importance of integrating preoperative inflammatory marker evaluation and microbiome assessment into routine care for patients undergoing the modified Whipple procedure. This approach mirrors the practice observed in cardiovascular surgeries, where routine blood biomarker assessments are utilized to predict and improve postoperative outcomes [31]. Similarly, the incorporation of preoperative assessments aligns with the role of imaging techniques, which have become indispensable in surgical oncology for planning and optimizing interventions [32]. Furthermore, it reflects the broader trend of personalized medicine, where multidisciplinary approaches are tailored to enhance the management of complex conditions and improve patient-specific outcomes [33].

Elevated CRP, IL-6, and procalcitonin levels may signal the need for enhanced postoperative monitoring and infection risk management. Additionally, optimizing comorbidities such as diabetes preoperatively could significantly reduce complication rates.

Patients with both dysbiosis and high inflammation had the highest complication rates (60%), emphasizing the synergistic relationship between these factors. Prior studies [34] have demonstrated how microbiome disruption exacerbates inflammation through mechanisms like gut barrier dysfunction and endotoxemia. Our findings support these observations and suggest that interventions targeting inflammation and microbiome health—such as prebiotics, probiotics, or fecal microbiota transplantation—warrant further investigation.

Future perspectives should also consider the integration of advanced microbiome sequencing technologies and machine learning algorithms to identify at-risk patients. These tools could stratify patients based on specific microbiome patterns and inflammatory profiles, enabling clinicians to implement preoperative and postoperative interventions more effectively. Moreover, prospective multicenter trials are needed to validate these findings across diverse populations and institutional protocols. Expanding the scope of research to include dynamic changes in the microbiome and inflammatory markers throughout the perioperative period could further elucidate their roles in surgical outcomes.

### Strengths and Limitations

This study presents a comprehensive and multifaceted analysis of clinical and immunological factors associated with perioperative complications in patients undergoing the modified Whipple procedure. The study offers novel insights into how systemic inflammation and immune responses may influence postoperative outcomes by integrating a range of immunological markers, such as CRP, IL-6, procalcitonin, fibrinogen, and D-dimer. Including these biomarkers provides a fresh perspective on predicting complications, which is underexplored in pancreatic surgery. Furthermore, the study benefits from a substantial sample size of 123 patients collected over ten years in a clinical setting, increasing the findings’ robustness. Its focus on real-world clinical data enhances its relevance and applicability for improving patient care and outcomes in similar surgical populations.

This study presents several limitations that must be acknowledged. As a retrospective analysis, it is subject to inherent biases, including the potential for incomplete data and the inability to control all confounding variables. The study’s single-center nature may also limit its findings’ generalizability to other institutions with differing patient populations or surgical protocols. Additionally, the lack of prospective data collection restricts the ability to establish causal relationships between the identified risk factors and the observed complications. While stringent inclusion and exclusion criteria were applied to define a homogeneous study population, this approach may have introduced biases by excluding patients with recent antibiotic or probiotic use, systemic infections, or prior abdominal surgeries. Furthermore, preoperative factors such as dietary habits and lifestyle, which are known to influence gut microbiota composition, were not assessed. Similarly, the analysis did not include intraoperative variables, such as blood loss, surgical duration, and anesthesia techniques, which are well-documented contributors to systemic inflammation and postoperative morbidity. Genetic predispositions, previously linked to variations in surgical outcomes [35], and intraoperative variables, such as blood loss and surgery duration, which are well-established contributors to postoperative morbidity [36], remain unexplored. Including these factors could significantly enhance the predictive model and improve its relevance and utility in clinical practice. Addressing these factors in future research would provide a more comprehensive understanding of the complex interplay between microbiota, inflammation, and surgical outcomes, ultimately enhancing the predictive model’s clinical relevance and utility.

## 5. Conclusions

This study demonstrates that microbiome dysbiosis and elevated inflammatory markers are strongly associated with perioperative complications following the modified Whipple procedure for pancreatic head cancer. There is a notable synergistic effect between these factors. Complications such as pancreatic fistula, delayed gastric emptying, and infections were significantly linked to systemic inflammation and gut microbiome imbalances. These findings suggest that incorporating these biomarkers into preoperative risk assessments and addressing comorbidities like diabetes, inflammation, and microbiome health could improve patient outcomes and reduce complication rates.

## Figures and Tables

**Table 1:** Demographic and clinical characteristics of patients undergoing pylorus-preserving pancreaticoduodenectomy.

Characteristic	Group A (*n* = 51)	Group B (*n* = 72)	*p*-Value
Age (years) ^(a)^	65.3 ± 8.1	62.7 ± 7.9	0.12
Sex (M/F) ^(b)^	30/21 (58.8%/41.2%)	43/29 (59.7%/40.3%)	0.91
MI (kg/m^2^) ^(a)^	6.4 ± 3.5	5.8 ± 3.2	0.28
Diabetes Mellitus ^(b)^	22 (43.1%)	15 (20.8%)	0.02 *
Cardiovascular Disease ^(b)^	18 (35.3%)	10 (13.9%)	0.01 *
Average hospital stay (days) ^(a)^	18.4 ± 4.6	10.2 ± 2.7	<0.001 *

(*) statistical significance, *p* < 0.05; ^(a)^ SD± Mean; ^(b)^ Percentage.

**Table 2:** Distribution of perioperative complications in Group A (*n* = 51).

Complication	Group A: *n* (%)
Pancreatic fistula	24 (47.0%)
Delayed gastric emptying	11 (21.6%)
Postoperative infections	9 (17.6%)
Intraoperative hemorrhage	7 (13.7%)
Biliary fistula	3 (5.9%)
Intestinal fistula	2 (3.9%)
Intestinal obstruction	4 (7.8%)
Abdominal wall herniation	1 (1.9%)

**Table 3:** Microbiome Composition in Groups A and B.

Bacterial Type	Group A (*n* = 51) (%)	Group B (*n* = 72) (%)	*p*-Value
*Lactobacillus*	203 ± 5.4	3.8 ± 7.2	<0.001 *
*Bifidobacterium*	8.7 ± 4.6	32.4 ± 6.1	0.001 *
*Enterobacteriaceae*	40.5 ± 6.7	20.8 ± 5.5	<0.001 *
*Clostridioides difficile*	15.2 ± 3.9	6.4 ± 2.2	0.02 *
Microbial Diversity Index	1.8 ± 0.4	3.2 ± 0.6	0.001 *

(*) statistical significance, *p* < 0.05.

**Table 4:** Inflammatory markers by gut microbiome status.

Marker	With Dysbiosis (*n* = 64)	Without Dysbiosis (*n* = 59)	*p*-Value
CRP (mg/L)	68.4 ± 12.7	30.5 ± 7.2	<0.001 *
IL-6 (pg/mL)	94.3 ± 13.1	2.8 ± 8.9	<0.001 *
Procalcitonin (ng/mL)	2.9 ± 0.9	1.1 ± 0.3	<0.001 *
Fibrinogen (mg/dL)	520 ± 85	375 ± 60	<0.001 *
D-dimer (ng/mL)	910 ± 220	510 ± 130	<0.001 *

(*) statistical significance, *p* < 0.05.

**Table 5:** Complications by inflammatory marker levels.

Marker	Category	Complication Rate (%)	*p*-Value
CRP (mg/L)	<40 (*n* = 68)	22%	<0.001 *
	≥40 (*n* = 55)	0%	
IL-6 (pg/mL)	<30 (*n* = 61)	18%	<0.001 *
	≥30 (*n* = 62)	56%	
Procalcitonin (ng/mL)	<0.5 (*n* = 49)	15%	<0.001 *
	≥0.5 (*n* = 74)	58%	
Fibrinogen (mg/dL)	<400 (*n* = 69)	20%	<0.001 *
	≥400 (*n* = 54)	48%	
D-dimer (ng/mL)	<500 (*n* = 89)	18%	<0.001 *
	≥500 (*n* = 34)	45%	

*n* indicates the number of patients in each category. Percentages represent the proportion of patients within each category who experienced complications. Patients were divided based on marker levels, with thresholds determined by clinical relevance. Statistical significance is denoted by an asterisk (*), with *p* < 0.05.

**Table 6:** Relationship between gut microbiome, inflammatory markers, and complications.

Group	CRP (mg/L)	IL-6 (pg/mL)	Complication Rate (%)	*p*-Value
Dysbiosis + High Inflammation (*n* = 40)	72.3 ± 11.8	96.4 ± 14.2	60%	<0.001 *
Dysbiosis + Low Inflammation (*n* = 22)	40.5 ± 8.9	48.3 ± 9.1	30%	0.002 *
No Dysbiosis + High Inflammation (*n* = 32)	35.2 ± 7.5	45.8 ± 8.2	25%	0.01 *
No Dysbiosis + Low Inflammation (*n* = 29)	20.1 ± 6.4	12.5 ± 5.8	10%	<0.01 *

*n* represents the number of patients in each category. Dysbiosis was determined by microbiome composition, while inflammation was classified as high or low based on predefined thresholds for CRP, IL-6, and procalcitonin levels. Statistical significance is denoted by an asterisk (*), with *p* < 0.05.

**Table 7:** Logistic regression analysis of the combined effects of dysbiosis and inflammation on perioperative complications.

Variable	Odds Ratio (OR)	95% CI	*p*-Value
Dysbiosis + High Inflammation	7.4	4.0–13.8	<0.001
Dysbiosis + Low Inflammation	2.9	1.5–5.8	0.01
No Dysbiosis + High Inflammation	3.2	1.8–5.7	0.003
No Dysbiosis + Low Inflammation	Reference	N/A	N/A

**Table 8:** Multivariate logistic regression analysis identifying independent predictors of perioperative complications.

Variable	Odds Ratio (OR)	95% Confidence Interval (CI)	*p*-Value
Diabetes Mellitus	2.3	1.2–4.5	0.01 *
Cardiovascular Disease	1.8	0.9–3.6	0.07 *
IL-6 ≥ 30 pg/mL	4.5	2.4–8.7	<0.001 *
CRP ≥ 40 mg/L	3.7	1.9–7.1	0.002 *
Procalcitonin ≥ 0.5 ng/mL	3.2	1.6–6.3	<0.001 *
Fibrinogen ≥ 400 mg/dL	2.8	1.4–5.6	0.01 *
Shannon Index < 2	5.4	2.9–10.0	<0.001 *
*Lactobacillus* < 25%	3.9	2.1–7.2	<0.001 *
*Enterobacteriaceae* > 30%	3.6	1.8–6.9	0.003 *

(*) statistical significance, *p* < 0.05.

## Data Availability

The datasets used and analyzed during the current study are available from the first author.
